# Health system characteristics and evidence-based asthma care

**DOI:** 10.3389/falgy.2025.1528526

**Published:** 2025-02-24

**Authors:** Alice L. Crawford, Gareth H. Jones, Jane Scullion, Dermot Ryan, John D. Blakey

**Affiliations:** ^1^ Department of Respiratory Medicine, Sir Charles Gairdner Hospital, Perth, WA, Australia; ^2^ Medical School, Curtin University, Perth, WA, Australia; ^3^ Department of Respiratory Medicine, Royal Liverpool and Broadgreen University Hospitals NHS Trust, Liverpool, United Kingdom; ^4^ Respiratory Medicine, University Hospitals of Leicester, Leicester, United Kingdom; ^5^ Usher Institute, University of Edinburgh, Edinburgh, United Kingdom

**Keywords:** asthma, health system, quality of care, best practice, model of care

## Abstract

Asthma is a common and complex syndrome, and a major cause of morbidity and healthcare costs. Clinicians have an array of evidence-based investigations and effective interventions at their disposal, but outcomes have not improved as much as trial evidence would suggest they could. This article discusses drivers behind this discrepancy using illustrative examples to highlight information gaps and barriers that impair the delivery of community and emergency asthma care and appropriate referral to specialist asthma services. It highlights organizational issues in the current system that lead to disjointed care that varies in quality. It also explores problems such as the adequacy of training for healthcare professionals, divergence from best practice guidance, and an acceptance amongst patients and practitioners of poor asthma control. This, along with inherent problems in the diagnosis of this heterogeneous disease, facilitates and perpetuates suboptimal care and outcomes. To help address the outcome gap, we discuss the potential for relatively simple, achievable and cost-effective actions that could potentially be taken by clinicians together with commissioners and managers of healthcare systems.

## Introduction

**Figure 1:**
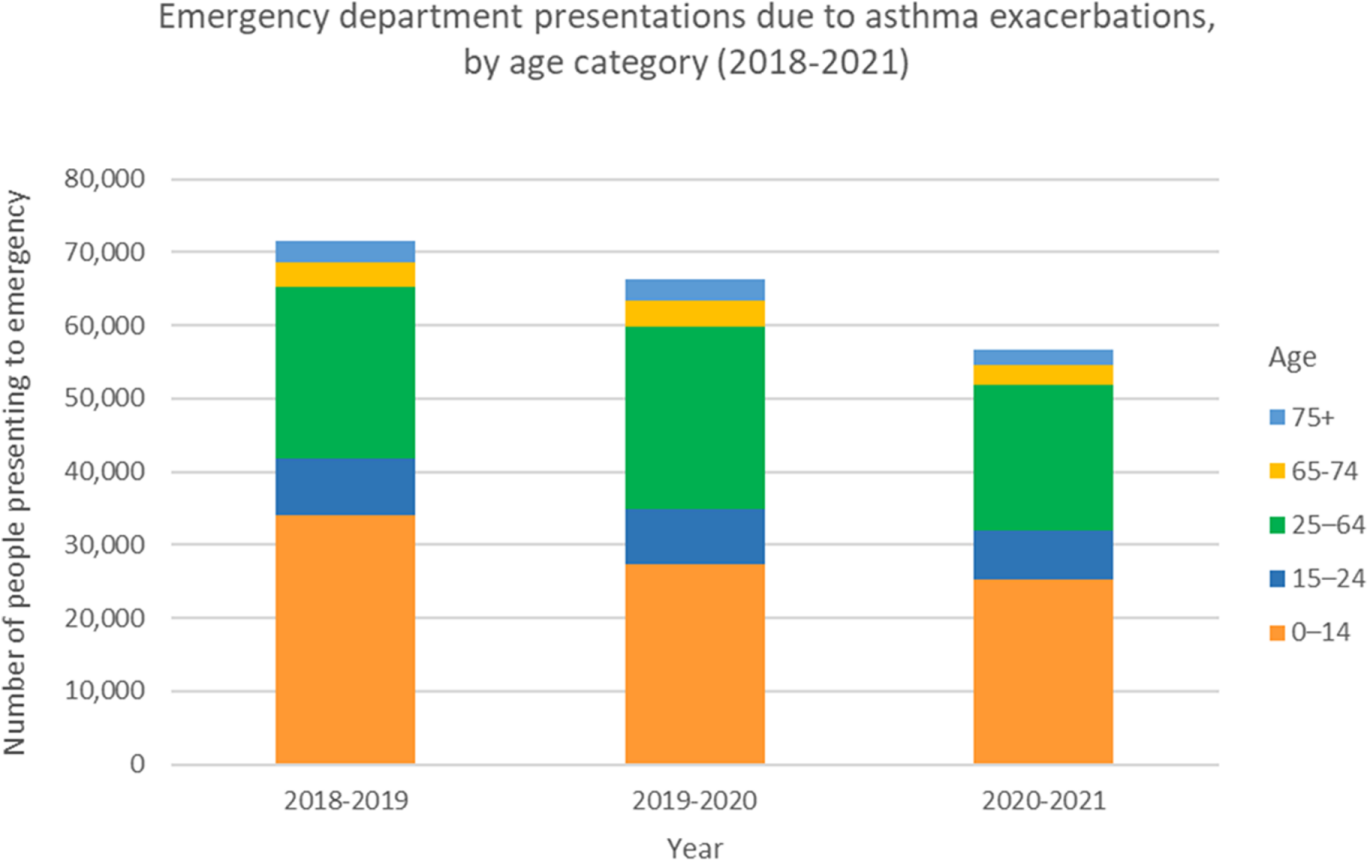
Emergency department presentations due to asthma exacerbations, by age category. Data from the Australian Institute of Health and Welfare, Asthma Tables 2023. Prior to the COVID-19 pandemic, the number of people presenting to emergency departments for asthma exacerbations across all ages peaked in 2018-2019 at 71,624 people. This represents a rate of 296.5 per 100,000 population. With strict public health measures during the pandemic in Australia, and relatively low rates of respiratory viruses, this number reduced to 56,587 people presenting to emergency for asthma exacerbations in 2020-2021. This represents a rate of 231.7 per 100,000 population.

Asthma is a common, chronic and multifactorial respiratory illness that affects people of all ages, ethnicities and regions worldwide. The burden of disease is manifested through a high level of unscheduled asthma admissions, emergency presentations (Figure 1) as well as a chronic symptom burden (1). In addition to direct annual costs of asthma, which amount to over £1 billion in the UK (2), the condition imposes similar additional indirect cost to society due to loss of productivity (3). The National Review of Asthma Deaths (NRAD) was initiated in response to persisting concerns over asthma mortality (4). NRAD was the largest study of its kind globally to date and found those who died were often not identified as being at high risk, and that uncontrolled asthma did not lead to scheduled specialist appointments (5). This is in keeping with other studies that suggest around 75% of hospital admissions and up to 90% of deaths related to asthma are considered preventable (6). This persisting, largely preventable, harm suggests we still need to make improvements in the deployment of existing efficacious interventions.

In this article, we discuss health system factors that can negatively impact asthma care. We use examples from England to highlight information gaps and other barriers that impair delivery of asthma care.

### Current state of asthma care

#### Asthma can be difficult to describe

Asthma has long been recognized as a heterogeneous syndrome with a variety of interacting comorbidities (7). Misdiagnosis of asthma is common (8, 9), with almost one third of people treated for asthma having no objective features of the disease in cross sectional studies. Worryingly many people are treated before a formal diagnosis is reached (10). Careful review to confirm a diagnosis is likely to be cost-effective (11). Processes of initial or reviewed diagnosis are made even more challenging by the variation in widely used asthma guidelines and handbooks on what constitutes asthma. For example, the global initiative for asthma (GINA) does not consider exhaled nitric oxide a diagnostic test, contrary to the British guidelines. Even those guidelines that provide a diagnostic algorithm however, do not always lead to patients receiving a definitive diagnosis (12).

People with similar clinical patterns of asthma can exhibit marked differences in their response to asthma medications (13). In recent years, there has been a move towards describing asthma more objectively in terms of the treatable traits present (14) and attempts to better define prevalent comorbidities (15, 16). However, we still lack a genuinely common terminology that is widely applied in primary care to specifically describe an individual's asthma. Applying a multifaceted series of labels may not be the most productive overall strategy: The improvements seen in cardiovascular outcomes in the past two decades relate to simple messages around intermediate phenotypes such as blood pressure and cholesterol rather than detailed description of the disease pattern in the arteries.

Increasing the difficulties in discussing individuals' asthma are issues around describing asthma control, severity and future risk (17). These overlapping terms are central to management guidelines (18, 19) but have been subject to competing definitions.

To contextualise issues with asthma services, we first consider the general way they are currently delivered. Although we appreciate this differs by country and region, there are core components and common problems.

#### Asthma care is disjointed and varies in quality

**Figure 2:**
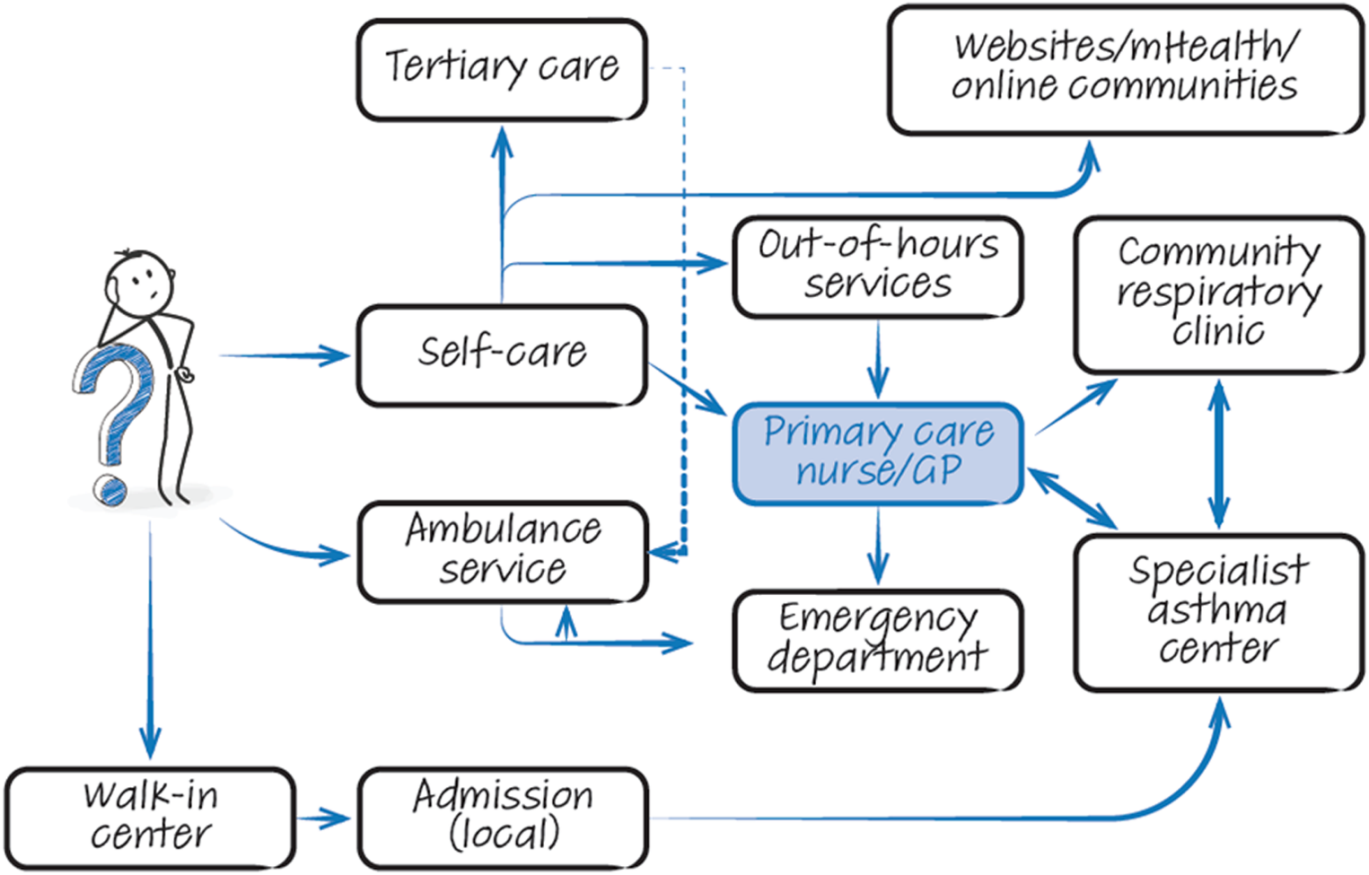
The complicated web of options. Graphical illustration of services available to patients with asthma when seeking treatment. Note that information flow is often suboptimal within this chaotic system. GP, general practitioner; mHealth, mobile health.

**Figure 3:**
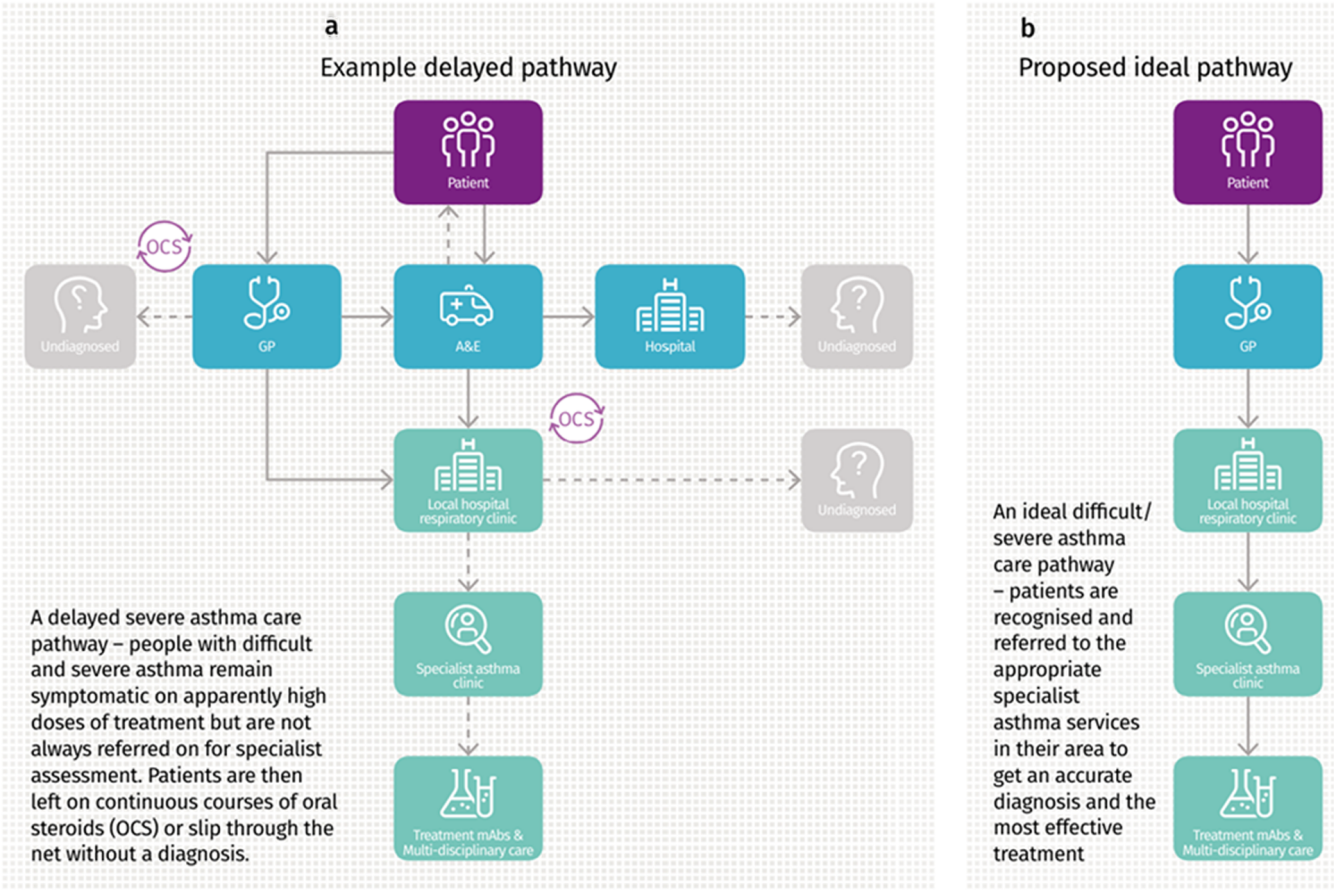
**(a,b)** Current delayed referral pathway for severe asthma, and the proposed ideal referral pathway. Image from Asthma UK “Slipping through the net: The reality facing patients with difficult and severe asthma” report, 2018. Asthma UK, 2018, Slipping through the net: The reality facing patients with difficult and severe asthma” accessed 02 May 2024 https://www.asthmaandlung.org.UK/sites/default/files/2023-03/auk-severe-asthma-gh-final.pdf.

People with asthma have a range of potential points of contact with healthcare professionals (HCPs) with overlapping roles (Figure 2). The patient journey through this web of services can be lengthy and difficult, particularly as they are often not designed with the involvement of experts and service users. Often patients attend through an emergency department, where inconsistent referral processes pose further challenges with expediting diagnosis and treatment optimisation (Figures 3a,b). Consensus documents where HCPs or people with asthma obtain standardized information on their local asthma services do not usually exist. There is also an asymmetry of information between patients and providers, meaning that individuals may not appreciate what good care looks like. This asymmetry is exemplified by the Living and Breathing Study, where patient satisfaction with their level of control fell significantly when they were shown Global Initiative for Asthma (GINA) targets for control (20). People with asthma in lower income areas often have lower health literacy (21) and are particularly at risk from not knowing what good care looks like. For those in lower income areas that try to progress through appropriate care pathways, they face a greater difficulty finding GP appointments (22), and relatively more financial stress travelling to have investigations or to specialist centres.

Lack of coordination and suboptimal patient outcomes may be expected when a providers' focus does not align with service user needs (see Table 1). These issues are exacerbated by lack of capacity, for example the UK has fewer doctors per capita (23) and shorter appointment times than countries with similar economic development (24, 25). Lower access to primary care for an asthma review is associated with an increased risk of future asthma-related emergency admissions (26). Insufficient topic specific knowledge also contributes, with most HCPs unable to correctly check inhaler technique (27, 28) and reporting a lack of adequate asthma-specific training (29, 30). The community asthma care landscape has shifted in the past three decades, with increasing delegation to appropriately trained respiratory nurses supported by national consensus guidelines. This approach can be successful but relies on both a high-quality training framework and clear delineation of how various healthcare providers should work together. The following sections discuss issues that in part arise from suboptimal training, delayed transitions of care, and uncertainty over who should be responsible for an aspect of care.

**Table 1:** Examples of potential misalignments between providers’ and service users’ targets in the management of asthma.

Provider		Targets/pressures	Consequence
Primary care	National helpline	Primary focus on whether to call an ambulance for breathlessness	No specific codes for asthma (personal communication with NHS 111 managers)
	General practice	Keep appointments brief	Insufficient time to assess
		Limit referrals (referrals not made even if criteria are met)	Insufficient time to educate. High-risk or comorbid patients not referred
		Prioritization of other conditions with more achievable targets	Persistence of uncontrolled disease
		Clinical pressure limits professional development time	Limited quality of annual asthma reviews
	Walk-in centres	Focus on addressing acute episodes	No changes to usual care
Secondary care	Ambulance service	Meeting high demand for transfer of patients	Limited communication of episode or treatment provided if not conveyed to emergency department
	Emergency department	Discharge within 4 h	Insufficient time to assess and educate. No follow-up arranged
	Inpatient care	Discharge due to bed capacity issues	Not cared for by respiratory medicine Discharged without action plan/follow-up
	Respiratory outpatients	18-week wait target New to follow-up ratio	Pathways are unclear or discourage referral. Patients not followed up

NHS, National Health Service (UK).

#### Best practice guidance is often not followed

A positive aspect of asthma management is the ready availability of clinical guidelines and quality standards. Guidelines vary (31) but have key elements in common regarding the fundamentals of care (12, 31, 32). The 2016 Annual Asthma Survey (33) found that one-third of people with asthma reported having received the core elements of guidelines, including an annual asthma review, a written asthma action plan and an inhaler technique check (34). Similar gaps in asthma care are seen in other high income countries (35, 36). OCS prescription in primary care remains relatively common, while combination inhaler use often appears to fall outside of the current guidelines (37, 38). Complicating this issue is the move toward basing guidelines almost exclusively on clinical trials [e.g., the National Institute for Health and Care Excellence (NICE)], so providing less guidance on best practice in common situations (e.g., pregnancy) or situations relevant to potential users [e.g., ambulance crews (39)]. Collaborative guideline development, such as the recent BTS-NICE-SIGN guidelines, reduce discrepancies and provide consistent national messaging (31, 35). Although promising, the extent of its impact will depend on uptake and implementation.

The discrepancy between ideal and observed care is striking for acute care. The Royal College of Emergency Medicine (RCEM) national audit of acute asthma (40) found that most unwell patients are not adequately assessed and a minority received appropriate corticosteroid therapy within 4 h of arrival at hospital. Few of those discharged directly from the emergency department had their inhaler technique checked and written discharge information was not consistently provided (40). Following an acute episode, a minority of individuals receive appropriate aftercare (34).

#### Patients and practitioners permit poor asthma control

Around half of the people with asthma have poor control when assessed using validated questionnaires (41–43). However, when asked more informally for their opinion, they and their physicians often significantly overestimate asthma control (44–47). This phenomenon has been observed for decades (48) and may relate to physicians using different criteria for assessing control than those in guidelines (49).

Compounding this issue are the perverse financial incentives in healthcare. Providers receive more income for a short admission for asthma than for seeing the patient in a clinic and preventing the admission. In practical terms, this carries the implication that disease control is less important than acute care.

**Figure 4:**
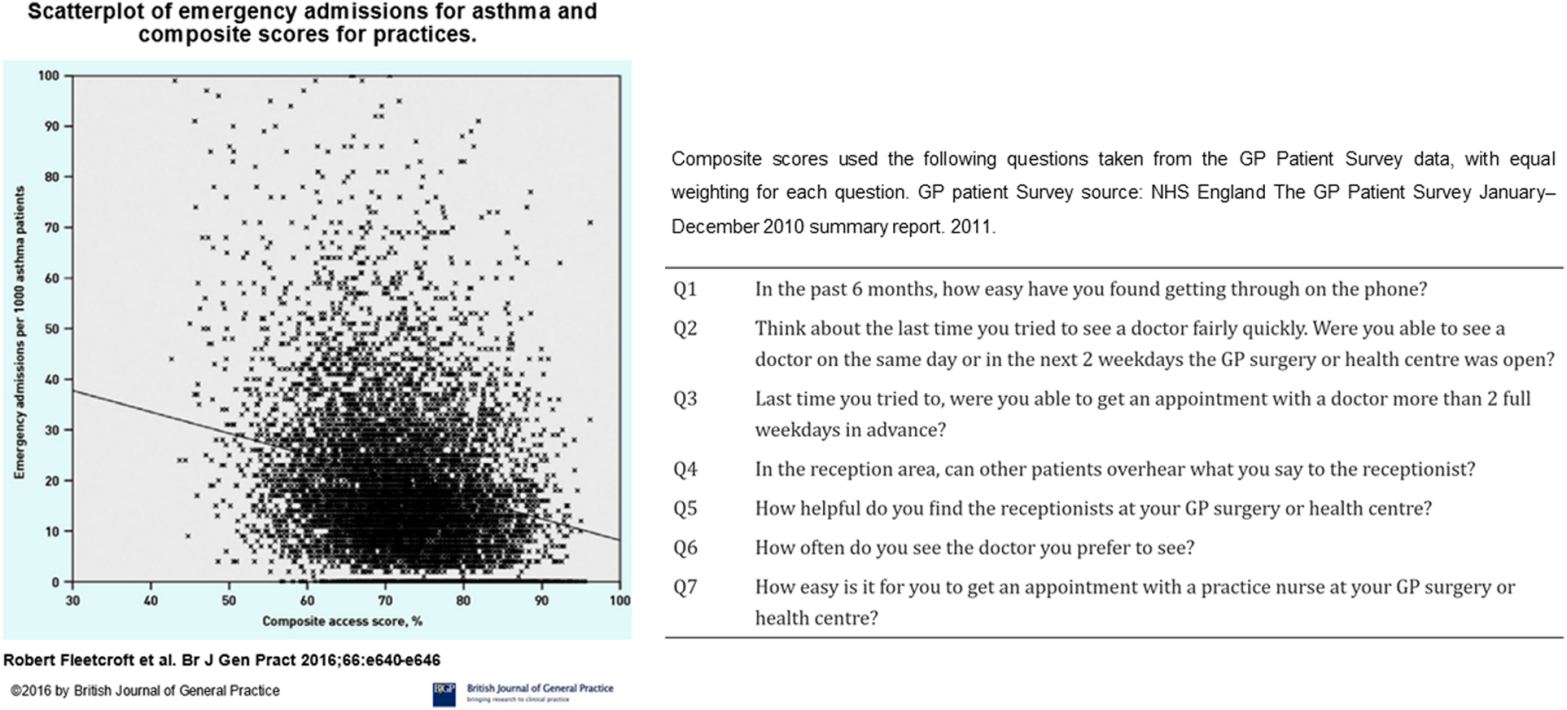
Scatterplot of emergency admissions for asthma against composite scores for access to asthma care. General practices with higher composite scores for access to care had fewer emergency admissions for acute asthma. Figure reproduced from: Fleetcroft R, Noble M, Martin A, Coombes E, Ford J, Steel N. Emergency hospital admissions for asthma and access to primary care: cross-sectional analysis. Br J Gen Pract. 2016 Sep;66(650):e640-6. doi: 10.3399/bjgp16X686089. Epub 2016 Jun 20. PMID: 27324628; PMCID: PMC5198697.

Accepting poor control means referral for specialist opinion is often delayed or not undertaken (50, 51). Referral thresholds are inconsistent, with primary and secondary care providers using different criteria to decide whether or not to refer (52). This pattern of referral has been highlighted by (52) a survey of clinicians that showed the delayed response allowed for the accrual of more harm (e.g., prednisolone courses), compared to the proactive approach advocated by guidelines. These problems are not confined to the UK (53). Expert review is therefore being delivered after personal suffering and negative economic impact (54) for patients who are often middle-aged (55), have acquired multiple comorbidities (55), and impaired quality of life (56–58),. Similarly, NRAD found many of those who died of asthma were not under the care of a Respiratory physician (5). For each of those who die, there are many more people in a cycle of acute illness, near-misses and oral steroid therapy (59) who have not been referred to secondary or tertiary care (34, 50). There have been laudable initiatives in some areas to encourage timely referrals (60). However, these have not been rolled out widely in a coordinated manner; there are geographical differences in observed outcomes (61), and markers of asthma care quality are also often discordant in primary and secondary care within areas (see Figure 4).

### Can systems be improved?

**Figure 5:**
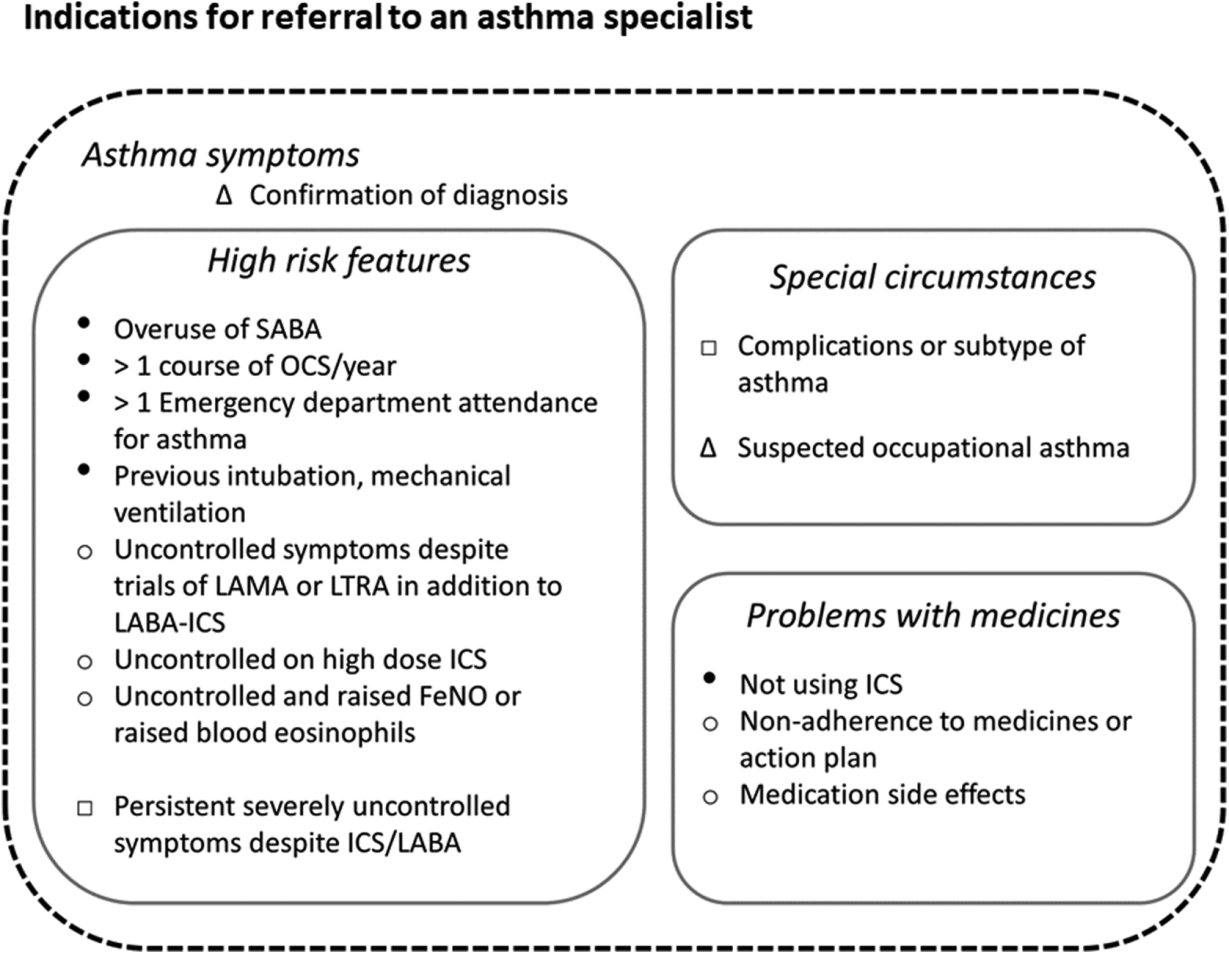
Key indications for referral to an asthma specialist. Indications for referral to an asthma specialist as described in the Global Initiative for Asthma (GINA), and British Thoracic Society/National Institute for Health and Care Excellence and Scottish Intercollegiate Guidelines Network (BTS/NICE/SIGN) guidelines. The urgency of referral is not specified in either document, however we highly recommend urgent referrals are made if any factors associated with increased risk of asthma-related mortality (indicated here with ●) are present. Legend: *Δ* documented in both GINA and BTS/NICE/SIGN guidelines. ○ Documented in BTS/NICE/SIGN guidelines. □ As per GINA guidelines. ● Factors associated with increased risk of asthma-related deaths as per GINA guidelines.

Improving asthma services is challenging in an environment where financial drivers can predominate over creating frameworks of excellence. Respiratory services generally are also under-funded compared with other disease areas. However, the foundations for quality improvement initiatives are already to hand. Demonstrating improvement with existing resources is also a pre-requisite of demanding further financial support from governments. Currently HCPs and clinicians can base their actions on frameworks for the diagnosis of asthma (62), individual-level management (19, 32), and system standards for primary, secondary and asthma specialist care (3, 63, 64). Tools to diagnose and monitor asthma, such as exhaled nitric oxide monitors, oscillometry, and smart inhaler devices, are becoming cheaper and more widely available. Despite this, few primary care practices – and indeed not all secondary care respiratory units- utilise them, often because of difficulties with reimbursement. Digital tools which identify high-risk patients are being more widely implemented primary care. One example is the Optimum Patient Care (OPC) service, a well-established UK audit scheme recently rolled out in Australia and designed to facilitate detection of suboptimal asthma control or treatment and facilitate onward specialist referral (65). Figure 5 illustrates the key indications for referral to an asthma specialist as mentioned in GINA and BTS/NICE/SIGN guidelines. This figure also highlights the factors associated with increased asthma-related deaths warranting urgent referral.

As the overwhelming majority of asthma care in the UK is delivered through the National Health Service (NHS), there is also clear scope to expand and act on regional and national data reporting (66), something that is challenging in other countries.

There is strong evidence that implementing consistent and widespread system changes can lead to marked improvement in clinical outcomes. The 10-year, country-wide program in Finland (67) was based on simple principles such as early diagnosis, proactive use of guideline-based treatment, education, tobacco avoidance, rehabilitation and research. It achieved its aim to lessen the burden of asthma on individuals and society, with a reduced number of days spent in hospital for asthma, and costs for both asthma care and disability benefits decreased. The Finnish programme viewed asthma as a public health problem that required public health solutions, and this is perhaps a key message for change. Improvements have not been sustained as there was no continuing investment – a common occurrence also in the UK.

Another potential solution to the variability in care provision is to systematically accredit clinics for the provision of airways disease care. This has been done in southern Sweden, an initiative that is now being rolled out across the country (68). Using a system of accredited centres also allows use of a standardized approach to diagnosis and treatment, potentially supported by clinical decision aids, as has been applied in the Netherlands for COPD (69). This also avoids the current difficulties potentially introduced by conflicts in treatment guidelines.

To produce positive change, any major issue must be broken into discrete targets that require well-defined actions by specific groups. We believe that these could potentially be summarized as action points (Table 2) for clinicians and healthcare management working collectively at local and regional levels.

**Table 2:** Potential action points to improve the asthma care system in England.

Domain	Target	Potential actions
Coordinated quality improvement	Ensuring lessons are learnt	NHS England could encourage NHS Trusts and CCGs to agree to national audited implementation of NRAD recommendations. Widespread implementation of standardised, accredited, guideline-based HCP respiratory training.
	Building on the National Asthma and COPD Audit Programme	Healthcare Quality Improvement Partnership could lead coordinated national cycle of planning, implementing and monitoring change in asthma care. Clinical interactions should not be a “tick box” exercise.
	Sharing tools for success	Potential use of Respiratory Futures (https://www.respiratoryfutures.org.uk/) as a platform to share successful protocols and templates (e.g., for run charts)
Common knowledge	Common language	British Thoracic Society Asthma Committee could lead work on an agreed glossary of asthma-related terms and definitions
	Explicit processes	Commissioned specialist asthma services might work with local CCGs to create public descriptions of regional asthma services and processes for referral
	Transparency in aims for asthma care	“Single source of truth” patient-facing description could be communicated by a body such as Asthma UK to describe what high-quality asthma care looks like, encouraging patient self-advocacy and education.
	Honesty in current performance	NHS England could publish provider performance against key quality metrics/standards
Innovative working	Primary prevention of attacks	CCGs could potentially implement increased use of risk assessment tools within electronic patient records
	Sharing difficult asthma expertise	Trusts might fund sessions for asthma teams from other hospitals to provide clinics
	Connecting specialists and patients	CCGs could increase the commissioning of community diagnostic and management clinics for asthma

CCGs, Clinical Commissioning Groups; NHS, National Health Service (UK); NRAD, National Review of Asthma Deaths.

### Working together

Success at scale will be achieved when invested parties work together in a coordinated fashion across provider boundaries. This was central to the progress made in Finland (67), but appears a barrier to improving outcomes in other countries such as the United States (70). Increased collaboration has been evident in the UK with the advent of severe asthma networks for adult services (71), with some progress through Primary Care Networks, and the National Asthma and COPD Audit Programme (NACAP).

Active patient involvement is important; however, we cannot rely on self-educated patients to drive change in a sub-optimally functioning system. Despite readily available quality asthma information, patients are facing an increasingly complex online environment with pervasive and sophisticated mis- and disinformation (72, 73), which may have deleterious social and health-impacts depending on the individual's online health literacy (74).

Efforts to initiate reform should avoid any perception of blaming colleagues and patients, which can fracture public confidence in HCPs. This is analogous to the car industry switching from criticizing “the nut behind the wheel” (in this case, “non-adherent patients” and “lazy GPs”) to collating better data and introducing subsequent safety advancements, such as anti-lock brakes and airbags, that require minimal user input and make allowances for real-world behaviours. Indeed, we have already taken first steps in this direction, as the concepts behind NRAD and NACAP are similar to the successful Fatality Analysis Reporting System in the car industry (75). This coordinated reporting should also facilitate the use of common language to describe types of asthma and common comorbidities.

### An explicit model of care

The management of asthma has largely been based on secondary prevention (17, 51), whereas other specialties have been successful in managing risk factors. For example, the rate of fatal heart attacks in England fell by about half between 2002 and 2010, (76) coinciding with widespread deployment of risk assessments. Emphasising both primary prevention and the use of specialised therapies to reduce harm has the potential to clarify the roles of primary and hospital providers. The majority of people with asthma have modifiable risk factors for future attacks, such as suboptimal inhaler technique (77). These factors could be identified and addressed in primary care in a more systematic fashion by leveraging electronic medical records, potentially with decision-support tools (78). However, decision-support tools are often ineffective if they are not incentivized (79). In the medium term a potentially promising avenue is the development of more community respiratory clinics that bring asthma specialists and quality assured diagnostics closer to patients (60, 80). Such services can improve cross-provider communication and facilitate training as Respiratory teams and GPs work alongside each other (81). Digital innovations such as remote sensors, telehealth solutions and mobile apps offer the opportunity to gather novel real-time data and radically redesign and decentralize asthma care in the future (82). This area is beyond the scope of discussion of this article. However, as there is no current consensus on the most effective or safest way to deploy such technology-based solutions, in the short term they risk introducing additional complexity and increased workload for healthcare providers (7, 83).

A key aspect requiring further study relates to understanding the barriers to referral to a hospital asthma clinic (7). However, two likely contributors to low referral rates could be relatively readily addressed. Firstly, the use of structured reviews can identify patients for whom control is not achieved (84), and can be undertaken by appropriately trained nursing staff when GP resources are stretched. Using standardized criteria should also facilitate the provision of appropriate details in a referral (85), including information such as prescription fulfilment rates that may not be accessible from secondary care. Secondly, systematic assessment at a dedicated severe asthma centre has been shown to improve quality of life and asthma control whilst reducing primary care/emergency department visits, hospital admissions and OCS use (86). It should be straightforward to create an online resource that ensures that GPs and, crucially, patients know where their nearest centre is (71)—indeed respiratory care maps have been developed by the NHS but inexplicably abandoned. This will avoid the unnecessary delays and loss of user confidence that occurs when people with difficult-to-treat asthma are referred to general respiratory clinics, as around half of people in the UK appear to be (87).

### Transparency and accountability

In this article, we have highlighted issues in existing systems that could be addressed to achieve incremental improvements in the overall care of individuals with asthma. However, drivers need to be developed by those at the highest level of provider organisations to ensure that transparency and accountability are upheld. This could include incentives to ensure that improvements in minimum standards are consistently achieved, such as the accreditation and appropriate renumeration of community respiratory clinics, with providers being obliged to publish rates of concordance with standards and statistical process control charts replacing less informative red-amber-green ratings. Recurrent central data collection (such as those provided by NRAD, NACAP and the RCEM audit) and quality improvement activities are fundamental, as are clear routes to access the data collected and stored at great expense.

## Conclusion

Interventions for asthma that have proven effective in RCTs are readily available, and guidance for their use and for referral to specialist asthma care are described in well-recognized guidelines. Despite there being relatively “easy wins” to be had, most people with asthma have suboptimal symptom control, and many have burdensome disease yet face a lengthy wait for a systematic review, if they are referred at all. When interacting with healthcare services, people with asthma receive variable and often suboptimal care. We are therefore exposing many thousands of people to preventable harm. It is clear that in order to improve quality and patient outcomes, a change in culture is required that focuses on the basic elements of quality improvement and requires the coordinated activity of clinicians and organisational decision-makers. We have discussed issues and potential action points that appear achievable and inexpensive when compared against the potential savings that improved asthma outcomes would bring. These changes can be summarised as moving toward transparent, rule-based, coordinated healthcare.

## Data Availability

The original contributions presented in the study are included in the article/Supplementary Material, further inquiries can be directed to the corresponding author.
